# Visual integration across fixation: automatic processes are split but conscious processes remain unified in the split-brain

**DOI:** 10.3389/fnhum.2023.1278025

**Published:** 2023-11-10

**Authors:** Yair Pinto, Maria-Chiara Villa, Sabrina Siliquini, Gabriele Polonara, Claudia Passamonti, Simona Lattanzi, Nicoletta Foschi, Mara Fabri, Edward H. F. de Haan

**Affiliations:** ^1^Department of Psychology, University of Amsterdam, Amsterdam, Netherlands; ^2^Amsterdam Brain and Cognition (ABC) Center, University of Amsterdam, Amsterdam, Netherlands; ^3^Department of Psychology, University of Turin, Turin, Italy; ^4^Child Neuropsychiatry Unit, Marche Polytechnic University, Ancona, Italy; ^5^Department of Odontostomatologic and Specialized Clinical Sciences, Marche Polytechnic University, Ancona, Italy; ^6^Psychology Unit, Ospedali Riuniti, Ancona, Italy; ^7^Department of Experimental and Clinical Medicine, Marche Polytechnic University, Ancona, Italy; ^8^Epilepsy Center-Neurological Clinic, Azienda “Ospedali Riuniti”, Ancona, Italy; ^9^Donders Institute, Radboud University, Nijmegen, Netherlands; ^10^St. Hugh’s College, Oxford University, Oxford, United Kingdom

**Keywords:** split brain, interhemispheric transfer, consciousness, confidence, visual matching

## Abstract

The classic view holds that when “split-brain” patients are presented with an object in the right visual field, they will correctly identify it verbally and with the right hand. However, when the object is presented in the left visual field, the patient verbally states that he saw nothing but nevertheless identifies it accurately with the left hand. This interaction suggests that perception, recognition and responding are separated in the two isolated hemispheres. However, there is now accumulating evidence that this interaction is not absolute; for instance, split-brain patients are able to detect and localise stimuli anywhere in the visual field verbally and with either hand. In this study we set out to explore this cross-hemifield interaction in more detail with the split-brain patient DDC and carried out two experiments. The aim of these experiments is to unveil the unity of deliberate and automatic processing in the context of visual integration across hemispheres. Experiment 1 suggests that automatic processing is split in this context. In contrast, when the patient is forced to adopt a conscious, deliberate, approach, processing seemed to be unified across visual fields (and thus across hemispheres). First, we looked at the confidence that DDC has in his responses. The experiment involved a simultaneous “same” versus “different” matching task with two shapes presented either within one hemifield or across fixation. The results showed that we replicated the observation that split brain patients cannot match across fixation, but more interesting, that DDC was very confident in the across-fixation condition while performing at chance-level. On the basis of this result, we hypothesised a two-route explanation. In healthy subjects, the visual information from the two hemifields is integrated in an automatic, unconscious fashion via the intact splenium, and this route has been severed in DDC. However, we know from previous experiments that some transfer of information remains possible. We proposed that this second route (perhaps less visual; more symbolic) may become apparent when he is forced to use a deliberate, consciously controlled approach. In an experiment where he is informed, by a second stimulus presented in one hemifield, what to do with the first stimulus that was presented in the same or the opposite hemifield, we showed that there was indeed interhemispheric transfer of information. We suggest that this two-route model may help in clarifying some of the controversial issues in split-brain research.

## Highlights

- Two experiments with the split-brain patient DDC.
- Experiment I shows that DDC is very poor at matching two stimuli presented across fixation but he feels nevertheless very confident about his performance.
- Experiment II shows that DDC can combine information presented separately in his two hemifields when he is forced to carry out a task in a deliberate, sequential manner.
- We propose a two-route model for the interhemispheric transfer of visual information. The first route is fast, automatic and unconscious integration of the two hemifields via the splenium. The second is a slow deliberate, conscious integration process.

## Introduction

The corpus callosum is the main connection between the two hemispheres (e.g., Innocenti, 1986; Gazzaniga, 2000; Wahl et al., 2007). In “split-brain” patients, the corpus callosum has been surgically cut to alleviate severe epilepsy that does not respond to medication. This operation may cause a curious phenomenon. When a picture of an object is presented in the right visual hemifield, the patient responds correctly with the right hand and verbally. However, when the object is presented in the left hemifield the patient verbally states that he/she saw nothing but nevertheless identifies the object accurately with the left hand only, for instance, by making a drawing of the object (Gazzaniga et al., 1962; Gazzaniga, 1967, 1998; Sperry, 1968, 1984; Wolman, 2012). This is in agreement with the human anatomy; the right hemisphere receives visual input from the left hemifield and controls the left hand, and vice versa (Penfield and Boldrey, 1937; Cowey, 1979; Sakata and Taira, 1994). Moreover, the left hemisphere is generally the site of language processing (Ojemann et al., 1989; Vigneau et al., 2006). Thus, it seems that resection of the corpus callosum causes each hemisphere to gain its own conscious awareness (Sperry, 1984). The left hemisphere is only aware of the right hemifield and can demonstrate this through its control of the right hand and verbal capacities, while the right hemisphere is only aware of the left hemifield, which it expresses through its control of the left hand.

On closer examination, this classic ‘response x visual field’ interaction appears less than absolute. First, Sperry (1968) himself already concluded that there are clear exceptions. Second, there are a number of earlier studies that failed to observe this interaction and found that responding was well-above chance with both hands (e.g., Levy et al., 1972; Kingston, 1994; Corballis, 1995). More recently, we (Pinto et al., 2017a) performed a quantitative study into this interaction. Rather than relying on qualitative summaries, we employed a quantitative approach. For this goal we had a substantial number of trials in each condition, forced-choice responding, and a large number of different stimuli. Moreover, we employed advanced fixation control with an eye-tracker,. The response type (left hand, right hand or verbally) was varied systematically. We found, in two split-brain patients, that although visual field played a major role in most tasks, a ‘response type x visual field’ interaction was never observed. This result held across all tasks (detection, localization, orientation matching, labelling and visual matching), and all tested types of stimuli ([isoluminant] dots, simple shapes, oriented rectangles, pictures of objects). Performance was always well-above chance and comparable with respect to accuracy across the three response modalities. Similar observations were recently reported by de Haan et al. (2020a) for the detection and localisation of tactile stimulation. Nevertheless, the information transfer between the two hemispheres in split-brain patients remains controversial. For instance, Volz and Gazzaniga (2017) have suggested that these effects might be caused by confounds such as ipsilateral arm control and/or cross-cueing. Ipsilateral arm control can be disregarded as an alternative explanation. There is very little evidence supporting the suggestion that ipsilateral control can support fine-grained distal movements of the arm required for pointing. The latter suggestion, i.e., cross-cueing, refers to the possibility that one hemisphere can inform the other hemisphere via subtle cues, such as touching the other hand or even via movements of the tongue in the mouth. In response, we have argued that this is an unlikely explanation (Pinto et al., 2017b). The main point being the fact that split-brain patients have no reason to develop intricate cross-cueing strategies as they do not experience problems in everyday life; with naturally occurring head- and eye-movements both hemispheres are fully informed about the outside visual world. In addition, such cueing is very limited in information load, probably not more than 1 bit, and most tasks that we used (Pinto et al., 2017a) required a more extensive information transfer.

Another observation in split-brain patients, that is not contested, concerns the fact that they are very poor at matching stimuli that are presented across the point of fixation. For instance, if two shapes or pictures of objects are presented for a “same” versus “different” decision with one in each hemifield, patients perform at chance level. Taking these two strands of evidence together, Pinto et al. (2017c) suggested subsequently that, although matching across fixation is no longer possible and the corpus callosum is thus necessary for integration of visual information from the two hemifields, split-brain patients continue to function as having a single mind and are able to respond using all response modalities.

Obviously, there is a paradox here that requires further investigation. If the single mind has access to information from both hemispheres (or to put it differently while remaining closer to the data; if both hemispheres – each controlling one hand – have access to information from both hemispheres), how come that a split-brain patient cannot match stimuli across fixation? One possible factor that might be helpful here is the distinction between automatic processing that does not necessarily provokes, or relies on, a conscious experience versus conscious, deliberate processing. There is abundant evidence for the position that to a substantial extent cortical processing of visual information proceeds without conscious awareness (e.g., de Gelder et al., 2001; de Haan et al., 2021). Therefore, the first question that we address here is to what extent is a split-brain patient consciously aware of the visual information in a simple matching task. In order to so, we developed a simultaneous matching task with both stimuli presented within one hemifield (left or right) or across fixation with one stimulus in each hemifield and using confidence ratings as a proxy measure for conscious awareness.

## Methods

### Case description

Patient DDC is a classic “split-brain” patient who also participated in recent studies by Pinto et al. (2017a, 2020) and de Haan et al. (2020b, 2021). In order to treat his medication resistant epilepsy, his corpus callosum was completely removed and most of the anterior commissure. Note that other than the resection of the corpus callosum, DDC has no brain damage, and he falls within the normal IQ range. See Corballis et al. (2010), Pinto et al. (2020), and Pizzini et al. (2010) for detailed clinical and radiological descriptions of this patient.

### Tracking eye movements

In both experiments, we measured eye movements with an Eyelink 1,000 (SR research, Mississauga, Ontario, Canada). In Experiment 1, trials were excluded from further analysis if a saccade – with a horizontal amplitude larger than 40 pixels (40 pixels <1.31° of visual angle) – occurred, after the experimenter started the trial and the stimuli had disappeared, or if there were any missing eye positions in this interval. In Experiment 2, trials were excluded if a saccade – with a horizontal amplitude larger than 40 pixels (40 pixels <1.31° of visual angle) – occurred, after the experimenter started the trial and the first stimuli had disappeared, or after the experimenter continued the trial and the second stimuli had disappeared with a horizontal amplitude, or if there were any missing eye positions during these intervals.

This is a conservative measure of saccades (generally eye movements up to 2° of visual angle are considered micro-saccades). Finally, in both experiments, at the start of the trial, the absolute measured horizontal eye position had to be within 80 pixels of the objective horizontal center of the screen. Again a conservative measure, since the only item on the screen at the start of the trial is the fixation spot. It is quite unlikely, even difficult to fixate anywhere else than at this fixation spot.

### Statistics

In both Experiment 1 and Experiment 2, we employed permutation testing for statistical testing, and a significance level of 0.05.

## Experiment I: Same-different matching

### Materials and stimuli

**Figure 1:**
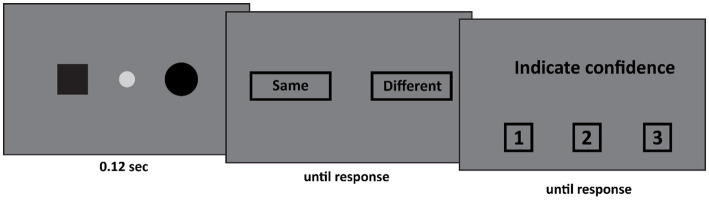
In Experiment 1 two stimuli appeared. Both stimuli either appeared in the left visual field, the right visual field, or around fixation (as depicted here). The participant first indicated whether both stimuli were the same. Subsequently he indicated his confidence in his judgment on a 3-point scale, where 1 indicated guess and 3 indicated certainty.

The set-up of Experiment 1 was as follows (see Figure 1). First, the patient fixated the fixation spot at the center of the screen (diameter: 0.44°, CIE x,y coordinates: 0.284, 0.311, luminance: 20.8 cd/m^2^) against a dark gray background (CIE x,y coordinates: 0.279, 0.304, luminance: 5.56 cd/m^2^). Subsequently, two shapes appeared. These shapes were identical on 50% of the trials, and different on the other 50%. The possible shapes were a black square (width and height: 1.11°), or a black circle (diameter: 1.28°) or a black triangle (width: 2.1°, height: 1.81°) appeared either 21° to the left or 7° to the right of the center of the fixation spot. All shapes were equally likely to appear. The two stimuli either appeared both in the left visual field (one stimulus 21° to the left of fixation, the other stimulus 7° to the left of fixation), around fixation (one stimulus 7° to the left of fixation, the other stimulus 7° to the right of fixation), or both in the right visual field (one stimulus 7° to the right of fixation, the other stimulus 21° to the right of fixation). This display was presented for 0.12 s. After this display a response screen was presented in which the patient indicated whether the two shapes were the same or different. This was followed by a confidence judgment where the patient indicated his confidence (from 1 to 3 where 1 indicated low confidence and 3 high confidence).

Stimuli were presented on a 24-in. monitor set to a resolution of 1920 × 1,080 at a refresh rate of 60 Hz controlled by a Dell Optiplex 760 computer (Dell, Dallas, TX) running Windows 8. The experiment was programmed in Matlab 7.7.0 (The Mathworks Company, Natick, MA) using the Psychophysics Toolbox routines (Brainard and Vision, 1997; Pelli, 1997). The patient was seated approximately 50 centimeters away from the screen. He rested his chin on a headrest to minimize head movements.

### Procedure

The trial was started manually by the experimenter. The experimenter, who could not see the stimulus screen, employed the eyelink to ensure that DDC fixated the center of the screen. Only if this was the case, the experimenter started the trial by pressing the space bar. Two stimuli appeared either within one hemifield, or across both hemifields. First DDC indicated whether both stimuli were the same or different. Subsequently he indicated his confidence in his judgment from 1 (guess) to 3 (certain). On half of the trials both stimuli appeared in one hemifield (equally often in the left and the right visual hemifield) and on the other half of the trials one stimulus appeared in each hemifield. Furthermore, on half of the trials both stimuli were the same, and on the other half of the trials both stimuli were different. All conditions were randomly intermixed. The experiment consisted of 3 blocks of 48 trials. DDC responded verbally (“same” vs. “different”) throughout the entire experiment.

### Results

**Figure 2:**
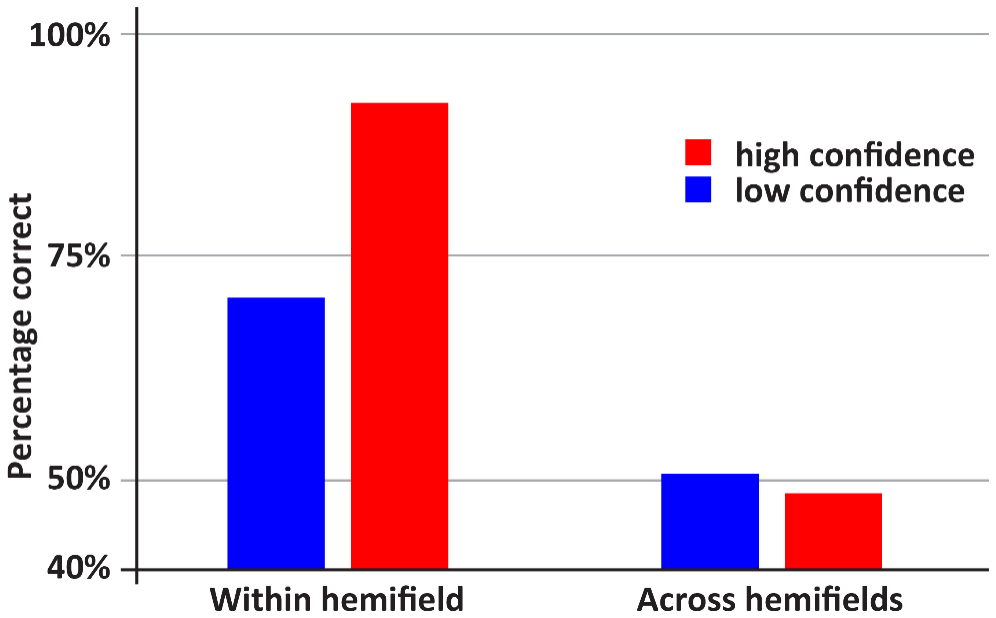
An overview of the results of Experiment 1. DDC was more accurate when the stimuli within one hemifield than when the stimuli appeared across hemifields. In the latter case, performance did not exceed random guessing. Moreover, only if both stimuli appeared within one hemifield, DDC was more accurate when he was more confident.

**Figure 3:**
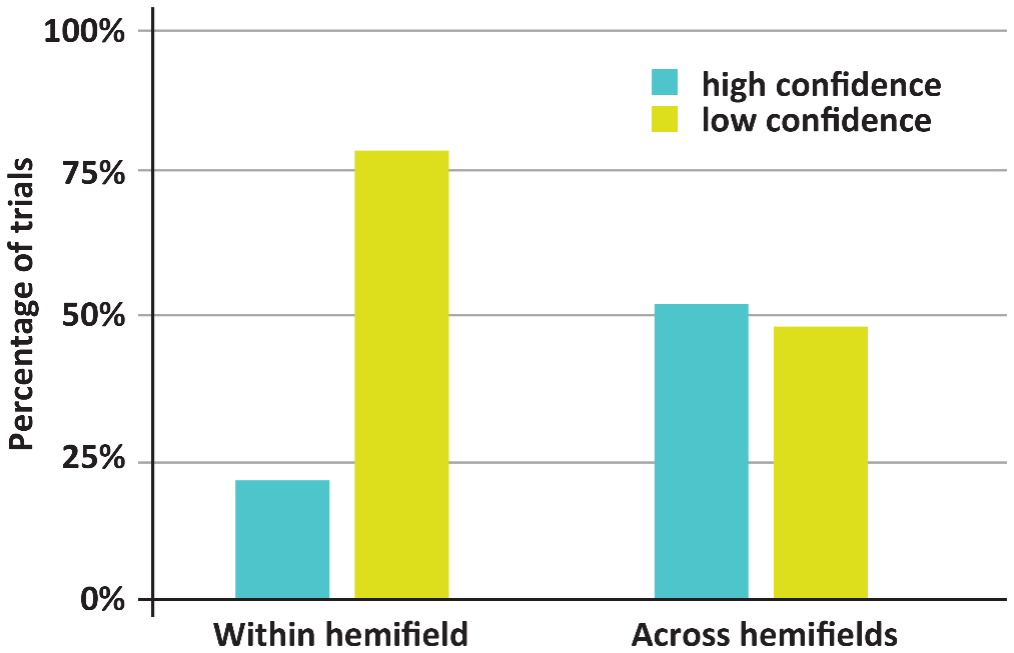
Incidence of low and high confidence trials depending on whether both stimuli appeared with or across hemifields. Remarkably, DDC was generally more confident when both stimuli appeared in different hemifields.

See Figures 2, 3 for an overview of the results of Experiment 1. If both stimuli appeared within one hemifield, DDC’s performance exceeded chance performance (*p* = 0.001), however when both stimuli appeared across fields, performance was not better than chance (*p* = 0.5). When both stimuli appeared in one hemifield, performance was better than chance, both on low confidence trials (*p* = 0.03) and high confidence trials (p = 0.001). However, when stimuli appeared across hemifields performance was not better than chance, both on low confidence and high confidence trials (ps > 0.43). Performance on high confidence trials was more accurate than on low confidence trials if both stimuli appeared within one hemifield (*p* < 0.05), but not when both stimuli appeared across hemifields (*p* = 0.73).

The main finding here is that despite worse (in fact, chance) performance when both stimuli appeared across hemifields rather than within one hemifield, the incidence of high confidence trials was higher in the former case. If both stimuli appeared across hemifields, DDC was highly confident on 52% of the trials. Yet, if both stimuli appeared within one hemifield DDC was highly confident on 21% of the trials. This difference was significant (*p* < 0.001). Note that in healthy adults the across hemifields condition is generally experienced as easier, since both stimuli appear more centrally in the visual field than if the stimuli appear within one hemifield. Remarkably, DDC had the same subjective impression – if stimuli appear centrally, the task ‘felt’ easier – despite the objective breakdown of his performance. In other words, DDC seemed to be completely unaware of his poor performance when the stimuli appeared in different visual fields. Anecdotally, during the experiment DDC remarked that he found the experiment much easier if the stimuli appeared around fixation (i.e., across visual hemifields).

### Discussion

First, this experiment replicated earlier studies (e.g., Pinto et al., 2017a) that had demonstrated that within-hemifield comparisons can be carried out accurately but that matching across the point of fixation is at chance-level. In addition, the accuracy of his within-hemifield matching is highly correlated with his confidence score, suggesting that he is consciously perceiving both stimuli and aware of the matching process. Thus, we conclude that he is not reacting in a reflexive fashion. However, and this is the most intriguing result, when the two stimuli were presented across hemifields, he was highly confident despite the fact his performance was at chance-level. In fact, he was more confident in the across- than in the within-hemifields conditions. The absence of a correlation between accuracy and confidence in the across-hemifields condition suggests that DDC is completely oblivious of his inability to compare stimuli that have been presented in his two hemifields.

There are, at least, two possible explanations for this observation. The first explanation is that the resection of the corpus callosum has resulted in an additional deficit affecting the introspective insight into his own functioning. In other words, it is a specific form of anosognosia. Although we cannot rule out this explanation, there is a second, perhaps more likely, explanation that concerns the possibility that automatic, unconscious visual integration across fixation is split but conscious processes remain unified in a split-brain patient. This hypothesis is based on the idea that the information from the two hemifields is integrated, via the corpus callosum, in an automatic, unconscious fashion in healthy observers. This fits the normal introspection; we do not experience a vertical line in the middle of our field of vision. It also fits with classic observation that split-brain patients generally feel unchanged (c.f. Bogen et al., 1965). Of course, in everyday life, they do not have a problem because they can make eye and head movement. As a result of his conviction that he perceives an integrated and complete visual field of vision, DDC feels confident in his visual matching but he is let down by the broken automatic integration of visual information across fixation.

If this explanation is correct, then it follows that the observation that a split-brain patient is able to respond accurately and confidently with both hands and verbally to stimuli presented anywhere in the visual field is based on a different, unified and perhaps conscious process. This process may be characterised as less visual, more symbolic in nature and may be communicated via subcortical pathways. In order to test this hypothesis, we developed the following experiment. It is aimed at the question whether he can combine visual information from the two hemifields in a conscious and deliberate fashion. Therefore, we adapted the first experiment in such a way that he now must process the two shapes separately.

## Experiment II: Integration across hemifields

### Materials and stimuli

**Figure 4:**
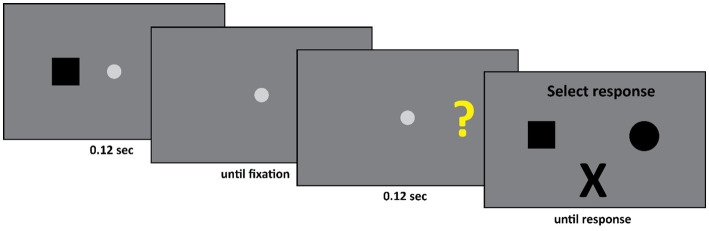
Initially either a square or a circle appeared in the left or the right visual field. Subsequently either a question mark or an “X” appeared, again either in the left or the right visual field. If the second stimulus was an “X” then the participant had to select an “X” as response. If the second stimulus was a question mark, then the participant had to select the first stimulus he saw. Thus, in this example, the square is the correct response.

The set-up of Experiment 2 was as follows (see Figure 4). First, the patient fixated the fixation spot diameter: 0.58° (CIE x,y coordinates: 0.284, 0.311, luminance: 20.8 cd/m^2^) at the center of the screen against a gray background (CIE x,y coordinates: 0.282, 0.309, luminance: 13 cd/m^2^). Subsequently, a black square (width and height: 1.11°) or a black circle (diameter: 1.28°) appeared either 13.4° to the left or 13.4° to the right of the center of the fixation spot. This display was presented for 0.12 s. Subsequently, only the fixation spot was presented until the experimenter pressed a spacebar. The experimenter did so when he ensured that the participant was fixating the center of the screen. The experimenter monitored the eye movements of the participant during the experiment; however, he could not see the stimulus screen. Finally, either a question mark (width and height: 1.11°, CIE x,y coordinates: 0.405, 0.521, luminance: 54.5 cd/m^2^) or an “X” (width and height: 1.11°, CIE x,y coordinates: 0.641, 0.341, luminance: 11.5 cd/m^2^) was presented either 13.4° to the left or 13.4° to the right of the center of the fixation spot. The final display was presented for 0.12 s. This was followed by the response screen where DDC could select one of three options – a square, a circle, or an “X”.

### Procedure

The trial was started manually by the experimenter. The experimenter, who could not see the stimulus screen, employed the eyelink to ensure that DDC fixated the center of the screen. Only if this was the case, the experimenter started the trial by pressing the space bar. Subsequently either a circle or square appeared for 0.12 s, either to the left or right of fixation, followed by a fixation spot at the center of the screen. At this point the experimenter again employed the eyelink to ensure that DDC fixated the center of the screen. Again, only if this was the case the experimenter continued the trial by pressing the space bar. Then either a question mark or an “X” appeared for 0.12 s, either to the left or right of fixation. Finally, the response display, consisting of a square, a circle and an “X” appeared and remained visible until DDC responded. The task of DDC was as follows. If the second stimulus was an “X” then he had to select an “X” from the response screen. However, if the second stimulus was a question mark, then he had to indicate what he had seen in the first display, by either selecting a square or a circle.

The experiment consisted of 4 blocks of 48 trials. There were four conditions, the shape either appeared in the left or the right visual field, followed by a second stimulus which again appeared either in the left or the right visual field – the second stimulus was either an “X” or a question mark. All conditions occurred equally often and were randomly intermixed throughout the experiment. The shape was equally likely to be a square or a circle, the second stimulus was equally likely to be an “X” or a question mark. Throughout the experiment DDC responded verbally.

### Results

In a control experiment we verified that DDC’s performance on directly comparing stimuli across the midline (same stimuli and same positions of the stimuli as in Experiment 2) did not exceed chance performance. This was true for all four stimuli. Thus, for example, DDC was no better than chance in indicating if the stimuli were the same or different if either two question marks or two squares appeared in LVF and RVF, or a question mark appeared in one visual field, and a square appeared in the other visual field. Moreover, DDC was highly accurate when he compared these stimuli to each other when they both appeared in one visual hemifield.

**Figure 5:**
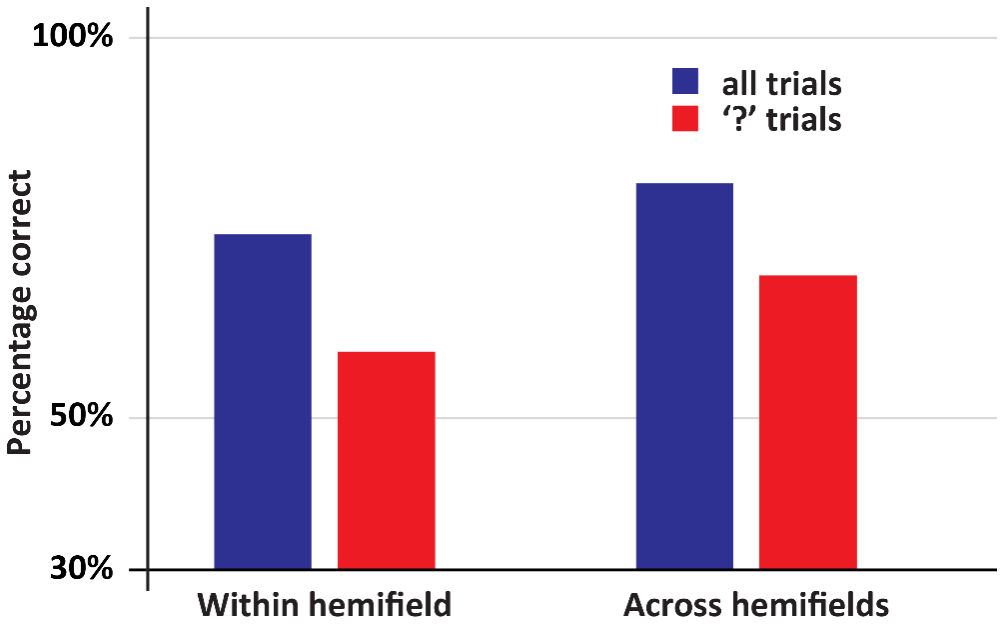
An overview of the results of Experiment 2. In this experiment DDC had to deliberately integrate information. Now his performance was at least as good in the across hemifields as in the within hemifield condition. Moreover, in all conditions his performance was well above chance.

The main result of Experiment 2 is that when the first stimulus and the second stimulus appeared in different fields, so that DDC had to make a deliberate delayed comparison across hemifields, he was highly accurate, see Figure 5 (Across hemifields accuracy: 80.8%, within hemifield accuracy: 74.2%. In both cases, performance was significantly better than chance, ps < 0.001. No significant difference between across and within hemifield accuracy, *p* = 0.19). Arguably, on the trials on which an “X” appeared as the second stimulus no across field integration was required, since DDC could simply ignore the first stimulus and select the “X” from the response set. Therefore, we performed the same analysis as before, but now we only included trials on which the second stimulus was a question mark. We again observed the same pattern as before. DDC was highly accurate even when stimuli appeared across hemifields, see Figure 5 (Across hemifields accuracy: 68.7%, within hemifield accuracy: 58.7%. In both cases performance was significantly better than chance, ps < 0.001. No significant difference was apparent between across and within hemifield accuracy, *p* = 0.29).

### Discussion

Crucially, in this experiment DDC’s was performing very well in all conditions. This was especially noteworthy when the second stimulus was a question mark, and the first and the second stimulus appeared in opposite hemifields. The main difference with experiment I is that he is now forced to pay active attention to the first stimulus: he might later on be asked whether it was a “□” or a “○”. This deliberate processing results in an almost perfect recollection of the first stimulus irrespective of whether this stimulus was presented in the same or the other hemifield. Thus, two hemispheres that are no longer connected via a corpus callosum interact to the degree that when one hemisphere is confronted with question mark, it can respond accurately to the question which shape had been presented to the other hemisphere.

## General discussion

In this study, we focussed on an important paradox observed in split-brain patients. On the one hand, split-brain patient cannot match stimuli across fixation, while on the other hand, it has now been established that these patients can respond accurately to stimuli anywhere in the visual field with either hand or verbally. Note: this paradox stands whatever explanation one wants to entertain to explain these effects.

Our starting point for addressing this issue was to look at the distinction between automatic processing that does not necessarily provoke, or rely on, a conscious experience versus conscious, deliberate processing. In Experiment 1, we used confidence ratings as a proxy for conscious awareness. In contrast to matching stimuli presented in the same hemifield, where his performance correlated with confidence (suggesting that he was well aware of what he was doing), there was no such correlation in the condition where he was asked to match across the point of fixation. In fact, while he performs at chance-level, he is extremely confident.

We hypothesised that in the healthy brain information from the two hemifields is integrated, via the corpus callosum, in an automatic, unconscious fashion. Due to the resection of the corpus callosum, this link has been severed in a split-brain patient. However, DDC has no reason to doubt his abilities in this respect. Just as in normal introspection (we experience an integrated field of vision), DDC also subjectively perceives an integrated and complete visual world under normal circumstances when he can move his head eyes. However, this automatic integration of visual information itself is blocked by the resection of the splenium. It is not immediately clear on the basis of what information he comes to the (often incorrect) response. Perhaps this is the result of a completion process across fixation (e.g., Sergent, 1988; McCarthy et al., 2006).

If this explanation is correct, then it follows that the observation that a split-brain patient is able to respond accurately and confidently with both hands and verbally to stimuli presented anywhere in the visual field is based on a different, unified and perhaps conscious process. We speculated that this process is characterised by less visual detail and more symbolic in nature, and that it might involve subcortical pathways. In order to test this hypothesis, we adapted the first experiment in such a way that he now has to process the two shapes separately. The results showed convincingly that under these experimental conditions, DDC was as accurate in combining information presented in two different hemifields as he as when the information was presented in the same hemifield.

It could be argued that the results of Experiment 2, where we aimed to force the split-brain patient to consciously integrate information across hemifields, were not caused by conscious effort, but by the delay between the first stimulus and the second stimulus. In other words, maybe the effect was due to changing the task from an immediate to a delayed matching task. Perhaps this temporal gap allowed for more cross-cueing for instance. However, note that such an explanation is unlikely to be true, as previous research has shown that a (partially) split-brain patient was not able to perform a delayed matching task either (Funnell et al., 2000). In other words, it seems that if a split-brain patient relies on automatic integration of information across visual fields, performance plummets, even if a temporal gap is added. The patient is only successful if he consciously integrates the visual information coming from both hemifields.

So, we propose a two-route explanation for these observations. In healthy subjects, the visual information from the two hemifields is integrated in an automatic, unconscious fashion via the intact splenium. It is fast and integrates detailed visual information but there is no possibility for introspection or control. This route has been blocked in a split-brain patient. However, the transfer of information remains possible when the patient is forced to use a deliberate, consciously controlled approach. The observation that he does not use this approach spontaneously is due to his false belief that he can rely on the first route in matching tasks. There are, at least, two questions that follow from this proposal. First, what is the nature of the information that is transferred via the second route? We suggest that it is more abstract, for instance the concept of “circle” or a “square”, or even the verbal label. As we used a response screen showing the different options and he responded verbally, we cannot distinguish between these possible options. The second question concerns the neural basis for this spared interhemispheric transfer. Several authors (Pinto et al., 2017c; Corballis et al., 2018; de Haan et al., 2020b) suggested that these effects could be the result of intact subcortical pathways. This is supported by the observation by Savazzi et al. (2007), who showed that the superior colliculus is likely to play a role in the visual transfer between the hemispheres. However, at present, it is not possible to exclude alternative explanations, such as quantum processing (e.g., Chalmers and McQueen, 2020) or complex cross-cueing. The latter is not very likely, as in the Experiment II it would have to be the responding hemisphere that has to probe the other hemisphere in half the trails (and not in the half). Such a deliberate and controlled strategy would be obvious to the patient. In our experience, patients have not commented on using such strategies.

## Conclusion

In two experiments, we found that automatic integration of visual information does not occur in the split-brain, yet deliberate integration remains possible. Moreover, the split-brain patient was unaware of this failure of automatic integration. Together these findings support the view that in a split-brain the conscious mind remains unified while automatic, unconscious processing is divided. We suggest that our proposal for a two-route model may help in clarifying some of the controversial issues in split-rain research.

## Data availability statement

The raw data supporting the conclusions of this article will be made available by the authors, without undue reservation.

## Ethics statement

Ethical approval was not required for the studies involving humans because This was part of standard research with the subjects. The studies were conducted in accordance with the local legislation and institutional requirements. Written informed consent for participation was not required from the participants or the participants’ legal guardians/next of kin in accordance with the national legislation and institutional requirements because this was part of standard research with the subjects.

## Author contributions

YP: Conceptualization, Methodology, Supervision, Writing – original draft, Writing – review & editing. M-CV: Writing – original draft, Writing – review & editing. SS: Writing – review & editing. GP: Writing – review & editing. CP: Writing – review & editing. SL: Writing – review & editing. NF: Writing – review & editing. EH: Methodology, Writing – original draft, Writing – review & editing.
